# Perinatal mental health around the world: priorities for research and service development in China

**DOI:** 10.1192/bji.2020.5

**Published:** 2020-08

**Authors:** Simone Eliane Schwank, Qiongjie Zhou, Yanling He, Ganesh Acharya

**Affiliations:** 1 PhD, MS, MA, Msc, Clinical psychologist, Department of Clinical Science, Intervention and Technology (CLINTEC), Karolinska Institutet, Stockholm, Sweden. Email: simone.schwank@ki.se; 2 MD, Specialist Obstetrician, Obstetrics and Gynaecology Hospital, Fudan University, Shanghai, China; 3 MD, MintMH, Director, Department of Psychiatric Epidemiology, Shanghai Mental Health Centre, Shanghai Jiao Tong University School of Medicine, China; 4 MD, PhD, FRCOG, Professor and Head, Division of Obstetrics and Gynaecology, Department of Clinical Science, Intervention and Technology (CLINTEC), Karolinska Institutet, Stockholm, Sweden

**Keywords:** Perinatal psychiatry, anxiety disorders, community mental health teams, psychiatric nursing, stigma and discrimination

## Abstract

China's healthcare is improving together with rapid economic growth. Yet, mental healthcare is lagging behind. Prevalence of perinatal depression is high among women of the one-child generation, but access to qualified care is limited. Chinese healthcare professionals, policy makers and patients alike express concerns about insufficient knowledge among the public as well as healthcare providers regarding mental disorders. There appears to be a general lack of help-seeking behaviour for mental disorders owing to perceived risk of social stigmatisation. Social support through family and friends, use of online resources and community healthcare services are preferred, rather than seeking help from mental health specialists.

It has been estimated that 39.6 million healthy life-years will be lost in China by 2025 because of mental illnesses (the estimate is based on disability adjusted life-years, or DALYs).^1^ The prevalence of mental disorders reported by the recent Chinese Mental Health Survey was higher than that reported in the previous two large-scale surveys from China and there are substantial gaps in the accessibility of care.^2^ The number of epidemiological studies on mental health in China is relatively small. Basic data on many mental disorders, populations and regions are still missing. There are so many knowledge gaps in this field that an attempt to fill even one or two of these gaps is valuable. The prevalence of perinatal depression in women with obstetric complications in China is estimated to be 10–15%^2^ and it is reported to be higher in women of the Chinese one-child generation compared with previous generations, particularly among women who give birth to a daughter.^3^ Perinatal depression appears to be more common in rural than in urban areas.^4^ Prevalence rates are highest in the rural areas’ prefecture-level cities (25.4%), lower in the provincial capitals (19.5%) and lowest in the municipalities directly under central government (12.9%).^5^ However, epidemiological research shows that women in metropolitan areas are especially at risk for developing common mental disorders.^6^

## Perinatal mental health in China

Perinatal mental disorders, including suicide, are emerging as a major cause of maternal mortality in both high- and low-income countries. In China, however, community members rarely consider mental illness a direct cause of suicide – it is typically seen as the result of family conflict or other social stressors. Exact national statistics on perinatal mental disorders are not available for China, but mental illness-related maternal mortality is reported to be negligible in Shanghai.^7^ The results of a recently published retrospective study on postpartum depression among Chinese women are consistent with previous findings that the majority (84%) of cases of postpartum depression occur within 6 weeks after childbirth and up to 94% within the first 4 months postpartum.^8^ This indicates higher risk for developing postpartum depression soon after childbirth, which is similar to Western countries, despite the tradition of familial support and rest after confinement, ‘doing the month’ (*zuo yue zi*). ‘Doing the month’ is family-based care during the period immediately after delivery, with the purpose of helping the mother to go through the physical and psychological adjustments. Its main components include avoiding cold, adequate rest, sufficient cleanliness, a balanced diet, pelvic recovery and nutrient supplements, believed to restore maternal postpartum health.^9^ In China, ‘doing the month’ has been considered to have both a positive and negative impact on maternal well-being.^10^

## Risk factors for perinatal depression in China

Women who gave birth to female infants, in the light of China's one-child policy, were at greater risk for postpartum depression.^11^ This is in contrast to studies conducted in Western societies, which did not find an association between fetal gender and postpartum psychology.^1^ China's gender preference has become a subject of substantial discussion since the implementation of the one-child policy in 1974. The one-child policy has been suggested to lead to stronger preference for males, and disappointment over the infant's gender is a significant risk factor for perinatal depression in China.^11^ Whether the recently introduced two-child birth control policy will have a positive effect on women's perinatal mental health remains to be seen.

Women experiencing postpartum depression report conflicts in attempting to conform to the Chinese traditional ideals perceived as necessary to maintain family harmony, while desiring to assert modern values such as individuality and independence. Living with extended family after childbirth is commonplace. It involves potential for relationship conflicts, as living with parents-in-law after childbirth has been described as a risk factor for perinatal depression among Chinese women.^10^ Higher prevalence of relationship conflicts was found in Chinese women with perinatal depression.^10^ Paternal perceptions of partner support and social integration reduce the risk for perinatal depression and are especially important in China, with its specific traditional extended-family dynamics. It has been reported that good postnatal care and ‘doing the month’ are protective factors against perinatal depression. Specific components of confinement practices might reduce psychological distress in Chinese mothers during the postpartum period, although the available evidence is inconsistent.^10^

## China's maternity healthcare system and perinatal mental health

China currently lacks national guidelines on perinatal mental healthcare and there is no systematic referral system for perinatal mental disorders,^12^ although recommendations have been made to improve mental health in the 2017–2020 national guidelines issued by the Chinese Ministry of Health and the 2030 Agenda for Sustainable Development. Shanghai is an exception, with perinatal guidelines issued by the Shanghai Centre for Women's and Children's Health in 2017 focusing on maternal mental health and recommendations for perinatal screening for mental health disorders.^12^ However, implementation of screening and treatment are yet to be established. Shanghai Centre for Women's and Children's Health promotes perinatal mental health information through a mobile phone app targeted at women with perinatal depression. Women visiting the antenatal clinic are screened for depression and anxiety. In Shanghai currently one second-tier maternity hospital and Fudan University Obstetrics and Gynaecology Hospital, which is a tertiary hospital, have routine antenatal screening for mental health problems, which was initiated in 2018. There is, however, a rising number of community health centres expanding their perinatal mental health services. In the screening programme, women with high scores on the 9-item Patient Health Questionnaire (PHQ-9) and/or the 7-item General Anxiety Disorder scale (GAD-7) are referred to multidisciplinary teams of clinical psychologists, psychiatrists, obstetricians and midwives. During the postpartum period women with moderate depression (Edinburgh Postnatal Depression Scale, EPDS, score >10) receive online services, including access to chat groups, information (videos, reading material) on mental health, breastfeeding and the mother–infant relationship, and online therapy. Women with major depressive disorder (EPDS score >15) or other severe mental disorders are referred to the Shanghai Mental Health Centre or secondary psychiatric hospitals. Similar services are also provided in several other institutions in China in different provinces.

## Mental health resources and service delivery

The World Health Organization's Mental Health Gap Action Programme (mhGAP) recommends psychological treatment as the first-line management for common non-psychotic mental disorders.^13^ However, resources and access to qualified mental healthcare services are limited in China, especially in rural areas.^14^ The majority of mental health professionals in China work in specialty psychiatric hospitals and the number of trained mental health professionals is inadequate to meet the patients’ needs and fill the treatment gap. Therefore, developing skilled human resources and enhancing the capacity of primary care services needs to be prioritised for delivering efficient mental healthcare at the community level.

## Traditions and societal pressures

In Chinese social settings, postpartum care is influenced by both traditional beliefs and contemporary healthcare practices.^9^ ‘Doing the month’ after childbirth involves a series of practices related to the maternal role, physical activity and a traditional diet, believed to restore maternal postpartum physical and mental health.^9^ Parents and parents-in-law encourage new mothers to adhere to traditional practices.^9^ Traditionally, mothers-in-law exercised significant power in Chinese households and had a major influence on the postpartum care of their daughters-in-law.^10^ In urban China there is an emerging trend of hiring a ‘maternity matron’ (specialised nurse) at home or ‘doing the month’ in a special maternity care centre. These services are expensive, but may reduce the women's burden of new motherhood. Parents and parents-in-law often share some of the cost. Family members and professionals provide compassionate care and boost the confidence of new mothers through their experience and skills, which has been generally perceived as a protective factor. However, with rapid cultural, economic and educational development in China, some urban and suburban women are now beginning to question such practices and to modify traditional postpartum care.^9^

## Help-seeking behaviour

A recent survey of Shanghai women found that over 70% did not seek help for perinatal mental health problems.^15^ A similar pattern is seen in the general population, and low help-seeking behaviour leading to lack of timely diagnosis and appropriate management of mental disorders may be associated with suicide.^16,17^ A majority (80%) of the Shanghai women participating in the survey would prefer information and counselling provided via online resources such as WeChat.^15^ Limited availability of affordable mental healthcare services, inadequate numbers of qualified and competent mental healthcare professionals, and social stigma associated with mental illness make women prefer online resources. Capacity-building through education and training of community physicians and other healthcare professionals as well as society as a whole is required to overcome the stigma and challenges associated with caring for women with perinatal mental disorders.

## Research priorities

Public health research is needed, including new epidemiological studies on perinatal depression in rural and urban China. Cost-effectiveness of treatments, including eHealth support, for common mental disorders during the perinatal period should be evaluated by properly designed studies. Studies focusing on women from the one-child generation are important because of the specific challenges associated with lack of siblings to rely on for social support and the known higher risk for developing mental disorders in this population. Research into the social support function of fathers and husbands is equally important in this regard. Further research regarding women's care-seeking behaviour for common mental disorders during the perinatal period is necessary to allocate resources in line with women's relevant needs.

## Conclusions

There seems to be an urgent need for improving mental health services in China, with a focus on perinatal mental healthcare. Epidemiological, clinical as well as health services implementation research on perinatal mental health need to be prioritised. Basic mental healthcare services should be available at community health centres to make them easily accessible and affordable. Educating and training more mental healthcare professionals, particularly those engaged in primary healthcare, retaining them in the healthcare system, and continuously improving their competencies and skills are essential to meet the mental healthcare needs of pregnant women and their families in China. Considering the very high demand and clear preference for online mental health services among the younger generation of pregnant women and new mothers, it would be appropriate to allocate resources to support the expansion of eHealth services and their integration into primary care in order to bridge the gap between the high prevalence of perinatal mental disorders and limited accessibility to mental healthcare services, especially for common mental disorders. Strengthening familial and social support systems could also have a synergistic positive effect on perinatal mental health.
